# Granular Jamming in Soft Robotics: Simulation Frameworks and Emerging Possibilities—Review

**DOI:** 10.3390/biomimetics11030193

**Published:** 2026-03-06

**Authors:** Stella Hrehova, Alexander Hošovský, Jozef Husár, Tibor Krenický

**Affiliations:** Faculty of Manufacturing Technologies with the Seat in Presov, Department of Industrial Informatics and Applied Mathematics, Technical University of Kosice, Bayerova 1, 080 01 Prešov, Slovakia; alexander.hosovsky@tuke.sk (A.H.); jozef.husar@tuke.sk (J.H.); tibor.krenicky@tuke.sk (T.K.)

**Keywords:** jamming, soft robotics, simulation frame

## Abstract

Soft robotics has become a dynamic field that emphasizes adaptability and safe interaction with complex environments. These structures utilize deformable materials and continuum mechanics to adapt their shape, absorb shocks, and perform tasks in unstructured environments. However, the design and optimization of these systems is challenging, primarily due to the nonlinear and discontinuous behavior of granular materials. In this paper, we address the role of simulation frames as an important tool for understanding, designing, and extending the functionality of software robotic devices utilizing granular jamming. The analysis suggests that DEM is essential for capturing particle-level mechanisms, while FEM is more effective for system-level optimization but tends to smooth out the transition of jamming. Hybrid FEM–DEM approaches provide the highest physical accuracy, albeit at an increased computational cost. Overall, the findings emphasize that the choice of framework must be application-oriented and that multiphysics coupling represents the future development. The review gives an up-do-date review of the simulation tools and approaches for granular-jamming-based systems with a specific focus on continuum arms with a granular-jamming-based central backbone. Such methods can be used for the optimization the back-bone geometry and its filling material (shape, porosity, granule size) with possible use in the real-time control of such arms.

## 1. Introduction

**Figure 1:**
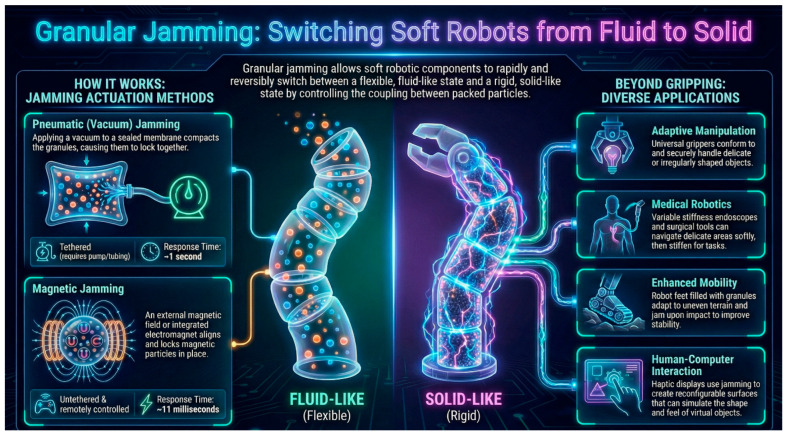
Schematic representation of the use of jamming in soft robotics.

Soft robotics has emerged in recent years as a promising research direction that responds to the growing need for robotic systems capable of safely and adaptively interacting with unstructured and dynamic environments. In the field of soft robotics, flexible and adaptable materials inspired by biological organisms are used instead of rigid links [1,2]. Unlike traditional rigid robots, soft robotics uses flexible materials and continuously deformable structures, which allows it to better absorb uncertainties, adapt to the shape of objects, and reduce the risk of damage when in contact with humans or the environment. Although this flexibility allows for safe interaction with the environment, the main challenge remains a variable stiffness mechanism that would allow robots to switch between a compliant state (for adaptation) and a rigid state (for force transmission) [3,4]. Most of these robots are equipped with mechanisms that allow adaptive changes in stiffness [5]. However, the challenge remains the ability to actively control the mechanical stiffness of the system so that it is possible to combine softness during interaction with the necessary stiffness for manipulation or force transmission. Damping is a popular and versatile soft robotic mechanism that enables the development of new systems that can achieve high stiffness variability with minimal volume variation [1]. It is defined as a physical process in which materials consisting of many smaller parts, such as grains or layers, change from a flexible to a rigid state [6]. Structures with the ability to actively control stiffness and damping enable adaptive and versatile interactions between machines and their environment. High stiffness enables precision, high forces, and speed. Conversely, low stiffness enables adaptability and safe interactions. Variable damping allows structures to respond to various external disturbances in unpredictable environments and enables a tunable frequency response [6]. The schematic use of jamming in soft robotics is presented in the following figure (Figure 1).

Among the common variable-stiffness methods for soft robotics, granular jamming stands out due to its fast response, high load-carrying capacity, and wide applicability, making it widely used in soft robotics research [7]. Structures based on this effect consist of granules enclosed in an elastomeric shell, with the stiffness controlled by vacuum pressure [5]. Granular jamming was the first type investigated in soft robotics applications and is still the most widely used [1]. Granular jamming exploits a reversible phase transition in granular materials [1,3,4,8]. This fundamental property allows a collection of grains to go from a pliable, fluid state to a stiff, solid state [1,3,4,9]. This transition, also referred to as the stiffness or jamming transition, is induced when individual grains are pressed together, usually by the action of a vacuum, while contained within an elastic membrane [3,10]. Granular jamming occupies an advantageous position in soft actuation because it offers a rapid change in stiffness with minimal volume change, providing a versatile actuation mechanism [1,3,4,8,9]. In the unjammed state, granular materials behave like liquids, allowing soft robotic structures to easily conform to objects or surfaces [3,10]. When a vacuum is applied, the resulting confining pressure compresses the particles, leading to mechanical interlocking and increased friction between the particles, which locks the assembly into a rigid, fixed configuration [3,4,9,10,11].

**Figure 2:**
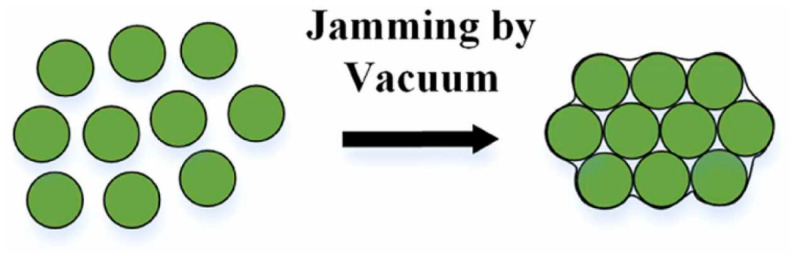
Schematic representation of the principle of granular jamming [10].

At reduced pressure in the sample (Figure 2), the pressure in the sample is close to the ambient pressure and the grains are free to move relative to each other. In this configuration, the system is in its flexible state, as it exhibits lower resistance to deformation (lower stiffness). When the pressure difference increases, the system is stiffer, as the grains begin to close together. In this new configuration, the sample exhibits greater resistance to deformation, as the internal reorganization of the grains requires greater force [12]. This principle has been successfully demonstrated in various soft robotic applications, especially in adaptive grippers, but also in the design of a diverse range of actuator configurations that enable the movement of worm-like and snake-like robots, soft robotic “paws”, and minimally invasive surgical instruments [13].

Although several mechanisms with variable stiffness have been developed in soft robotics, granular jamming occupies a distinct position due to its combination of mechanical performance, reversibility, and structural simplicity.

- Unlike phase-change materials, which rely on thermal transitions and therefore suffer from relatively slow response times and thermal management issues, granular disruption is typically pressure-driven and can achieve stiffness modulation within milliseconds.
- Unlike tendon-driven stiffening mechanisms, which increase stiffness through geometric constraint and active straining of structural elements, granular disruption enables volumetric stiffening throughout the structure rather than along predefined load paths.
- Compared to layered interference, which is often limited to planar or beam configurations and provides anisotropic stiffness primarily in bending, a granular interference offers nearly isotropic strengthening and can accommodate complex three-dimensional geometries.

Furthermore, granular systems exhibit fluid-like behavior in the unbound state, providing excellent conformability to irregular shapes before solidification, which is not fully achievable with layered or tendon approaches. It also enables high load-carrying capacity with a minimal overall volume change, which is advantageous for the integration of compact actuators.

Nevertheless, the behavior of systems with granular jamming is still only partially explored from both physical and engineering perspectives, mainly due to strong nonlinearity, discontinuities in contacts, and sensitivity to large numbers.

The significance of the presented paper lies in the fact that, based on selected literature sources with a focus on the last five years, it clearly identifies simulation frameworks with their advantages, limitations, and application possibilities in the field of soft robotics. It shows the shift from simple vacuum grippers to hybrid and multi-chamber systems, identifies knowledge gaps, and maps the deployment of jamming in various domains, which can strengthen the transition phase from empirical research to exact engineering approaches.

### Methodology

This paper systematically reviews the scientific literature on granular jamming in soft robotics, focusing on numerical simulation frameworks including the finite element method (FEM), discrete element method (DEM), and hybrid FEM–DEM approaches. The reviewed literature covers the period 2015–2025, with a greater emphasis on studies published in the last five years (2020–2025) to reflect the latest advances in modeling techniques and applications.

Relevant publications were identified using Elicit AI, an AI-powered academic research tool that enables the relevance-based retrieval of peer-reviewed literature across multiple databases. The search was conducted using combinations of keywords such as “granular jamming”, “soft robotics”, “FEM”, “DEM”, “hybrid simulation”, and “applications”, refined with logical operators to ensure comprehensive coverage.

Publications were included if they

- addressed granular jamming or jamming-based mechanisms in soft robotic systems,
- used FEM, DEM, or hybrid numerical modeling approaches,
- were published between 2015 and 2025,
- appeared in peer-reviewed journals or proceedings of peer-reviewed conferences.

**Figure 3:**
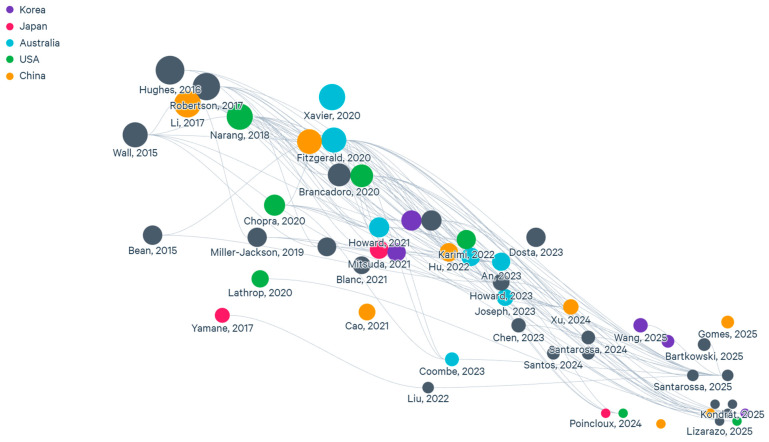
Map of literature sources.

Studies without a numerical component or without methodological details were excluded. The selection process consisted of three phases: an initial search, title and abstract screening, and full-text evaluation. After this process, 116 publications were retained for analysis. The selected studies were classified according to the simulation method used, physical phenomena modeled, application area, and validation strategy. A qualitative comparison was then performed to identify methodological trends, strengths and weaknesses of the individual approaches, and emerging research directions. Using Litmap AI, the following resource map (Figure 3) shows publications by year of publication, with the color representing the country of the first author. Only those resources with more than 5 publications are shown in color.

## 2. Principles of Jamming in Area of Soft Robotics

**Figure 4:**
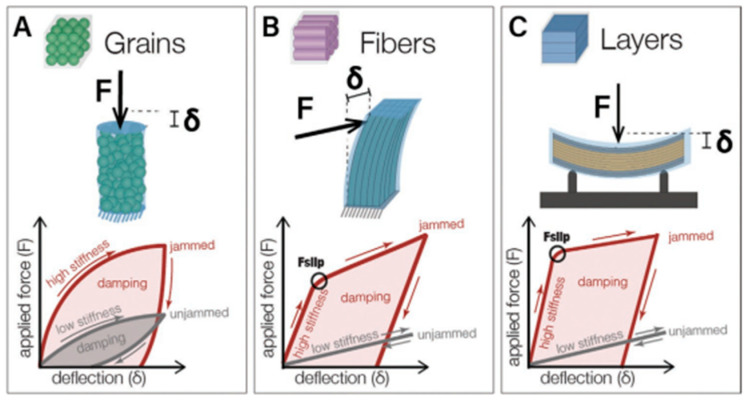
Schematic representation of individual types of jamming [7].

Although granular jamming is one of the most versatile techniques for achieving variable stiffness in the field of soft robotics [4,9,14], the literature reports other different types of jamming mechanisms used in this field [1]. These types are generally classified according to the material medium used to achieve the jamming transition (see Figure 4):

- Granular jamming: This mechanism involves the use of granular material (such as grains, coffee grounds, rice, plastic spheres, or glass beads) enclosed in a flexible, airtight membrane [1]. Stiffness is achieved by applying an external stress, usually vacuum pressure, which forces the grains into a solid state with high density and solidity through mechanical bonding and increased friction between the particles [4,7,12].
- Fiber jamming: These are fiber bundles where the change in stiffness is achieved by an interaction and friction between fibers when an external pressure or restraining mechanism is applied) [1,12,14,15].
- Layer jamming: In this method, the granular material is replaced by layers that stiffen when pressed against each other [6]. They change from a flexible state to a rigid state when external pressure is applied to them, which induces friction between the layers [1,12,14,16,17,18]. Layer-jamming systems are generally limited to planar structures.

Layered and fiber jamming typically require less volume and are lighter than granular jamming systems (advantage of their planar and long tube design), but their range of stiffness tuning and passive deformation without jamming is not as large because they do not behave like fluids in the unjammed state [1]. More detailed descriptions of each type can be found in the literature [7].

D. Tabular jamming: Tubular jamming produces a jamming effect similar to granular jamming, but positive pressure is used instead of vacuum and pressure; instead of inducing a vacuum to contract the structure surrounding the incoming particles, the tubular jamming method expands the particles within a fixed volume structure [18].

In the next section, we will focus on granular jamming.

### 2.1. Definition and Mechanism of Granular Jamming

Granular jamming is the most widely used and studied type of jamming [19]. It is a phenomenon in which a granular material placed inside a flexible non-porous membrane can reversibly transition from a liquid state with low stiffness to a solid state with high stiffness [3,20,21]. This transition is mainly induced by a negative pressure difference across the membrane (vacuum). When the internal pressure drops and the inner wall compresses the granular medium, the granular medium changes to a solid state. Conversely, when the internal pressure returns to normal atmosphere, the granular medium becomes liquid. This makes it much stiffer and able to retain its shape [7,22].

Two main types of grains are used for granular jamming [1]:

- natural (coffee, corn, gravel, rice, pepper, salt, sugar)
- artificial—including plastic, glass or rubber materials with spherical, cubic or cylindrical geometry.

Granular jamming can be divided into the following:

- Passive granular jamming: The stiffness is changed passively due to external pressure or deformation of the actuator itself, without the need for an external vacuum. The method consists of connecting a soft silicone rubber actuator and a particle cluster. Inflation of the soft actuator exerts pressure on the particle cluster, causing the particles to jam inside [23]. This is advantageous for soft actuators, as they always inflate. Inflation is thus used to exert external pressure on the particle cluster.
- Hydraulic granular jamming: Uses an incompressible fluid (deaerated water) instead of air to transmit pressure, allowing for miniaturization of the system [24].

The membrane is responsible for retaining the granular mass and creating an interface between the object and the grains, so the performance of the gripper largely depends on it, as the literature states that the membrane must be flexible, adapt to the objects, and withstand cycles of use without failure due to material fatigue [4]. The operating principle in soft robotics lies in the interaction between the granular medium and the elastic membrane:

- System composition: The system consists of a collection of independent solid particles (e.g., ground coffee, glass beads, sand, plastic granules) enclosed in a flexible, airtight membrane [10,13].
- Unjammed state: When the internal pressure in the membrane is close to the ambient atmospheric pressure, the particles are loosely arranged and exhibit low friction [4,9]. In this state, the material has low viscosity and low resistance to deformation, allowing the structure to passively conform to irregular objects [3].
- Initiation of jamming: The transition to the solid state is achieved by creating a vacuum (negative pressure) inside the membrane [1,3,11]. This pressure difference causes the external atmospheric pressure to compress the membrane inwards, thereby compacting the particles [4,10].
- Consolidation process: The pressure difference causes the external atmospheric pressure to push the membrane inwards, causing the particles to compact. This compaction drastically increases interparticle friction and the packing density (volume fraction), which is the ratio of the volume of the particles to the total volume of the system.
- Formation of force chains: Particles mechanically interlock (inter-lock) under compression and form so-called force chains. These chains transmit mechanical stress throughout the structure, giving it stability and a fixed modulus of elasticity.
- Gripping mechanisms: The total holding force is created by a combination of three factors: static friction on the surface, geometric interweaving, and suction, if the membrane creates an airtight seal around the object.
- Reversibility: The entire process is fully reversible [1,11]—after the pressure is restored, the mechanical bonds are released and the particles regain their original mobility.

#### Factors Affecting Jamming Performance

The efficiency and resulting stiffness (Young’s modulus) depend on several design parameters:

- Granule properties: The shape, size, and material of the particles determine the degree of friction and entanglement [4,13]. Soft particles (such as expanded polystyrene) can paradoxically lead to higher gripping forces due to the “squeezing effect” and better adaptation of the membrane to the object [3].
- Membrane material: The thickness and elasticity of the membrane affect not only the actuation force required for deformation but also the quality of the airtight seal [2,4].
- Vacuum level: The stiffness of a system typically increases linearly with an increasing pressure differential. This principle allows the design of universal grippers that can handle fragile or complex-shaped objects without the need for complex sensory equipment [2,4].

The grain size is considered the most important factor for the blocking property. In general, a smaller size is advantageous for good flow and adaptability, as well as high blocking force [3].

### 2.2. Simulation Frames

Simulating the interactions between the granulate and flexible membrane is considered a major challenge in soft robotics, as this relationship is highly nonlinear and difficult to capture with simple approximations [4]. The traditional experimental approach to the design of soft robotic systems with granular jamming is often time-consuming and expensive, as it requires repeated prototype fabrication and extensive testing for different configurations of granules, packaging materials, and pressure conditions. Moreover, experiments provide only a limited insight into the internal dynamics of the granular medium, which plays a crucial role in the jamming process. For this reason, numerical simulations become an indispensable tool for a deeper understanding of the mechanisms of granular jamming and for a systematic investigation of the behavior of these systems under a wide range of conditions. However, the simulation of granular-jamming systems in the context of soft robotics poses a significant challenge. Discrete approaches, such as the discrete element method, allow for the detailed capture of interactions between individual particles, but at the cost of high computational complexity. In contrast, continuous and approximate models provide more computationally efficient solutions but often fail to faithfully reproduce the micromechanisms leading to jamming. Hybrid simulation frameworks that combine the advantages of both approaches therefore appear to be a promising path towards realistic and, at the same time, practically applicable modeling.

In this context, simulations not only play the role of a validation tool but are increasingly becoming an active part of the design process. They allow for extensive parametric studies, testing new geometric and material configurations without the need for physical prototypes, and identifying design principles that would be difficult or impossible to discover experimentally. In addition, they open up the space for integration with machine learning and optimization methods, leading to the emergence of digital twins of soft robotic systems with granular jamming. Such approaches fundamentally expand the possibilities of using variable stiffness and represent an important step towards intelligent, adaptive, and efficiently designed soft robotic solutions.

Several software frameworks are available for the simulation of granular materials. All use the same principles of explicit time integration, with each time step consisting of three main steps: contact detection, interaction calculation, and integration of the equations of motion. However, there are significant algorithmic differences, such as the choice of contact models, particle and wall shapes, and data analysis methods [25].

(a) Finite Element Method (FEM): The FEM is primarily used to model membrane deformations and stress distributions throughout the system [9,26]. Advanced models (e.g., in ABAQUS software) use hyperelastic material models (such as Yeoh or Ogden models) to predict the mechanical behavior of soft actuators [5,27,28]. In the literature [16], the authors used FEM to predict the stiffness, damping, and kinematics of multilayer structures under both laminar (layered) and granular jamming. This approach was also used to verify the behavior of granular chains and their ability to resist bending moments [29].

Simulation tools include the following:

- COMSOL Multiphysics v. 6.4: The most frequently mentioned commercial tool. It is used for the following:
  - Modeling hollow membrane geometries and their adaptability (shell module) [2].
  - Analysis of magnetic fields and magnetization of composites for MGJ systems (AC/DC Module) [21,30,31].
  - Simulation of mechanical performance and deformation of Kirigami structures [27].
  - 3D simulation of material models (neo-Hookean) for compressible rings [32].
- ABAQUS: Prominent in the analysis of pneumatic structures and hollow bellows structures. Often combined with other tools for solving particle interaction problems. [27,28,29].
- LS-DYNA: Used to implement the proper constitutive equations for modeling cyclic loading of a granular core, which in this case is considered a continuum [5].

(b) Discrete Element Method (DEM): This framework is key to understanding particle-level interactions and the formation of force chains that transmit stress in a stuck state [7]. This method is currently a widely used approach to modeling granular processes in engineering and robotics [19]. The DEM is used to study the influence of the granule shape and size on grip stability and surface adaptability [4,22]. Open-source frameworks such as MercuryDPM or LIGGGHTS allow for the verification of contact models and simulation of bulk processes [4,25]. The traditional DEM algorithm focuses on rigid, unbreakable, spherical particles and uses force models for pairwise interactions with other particles and walls, as well as for external body forces. However, since its introduction, various extensions have been proposed [15].

DEM tools are key for simulating interactions between individual grains, their friction, and the formation of force chains.

- LIGGGHTS: A popular open-source DEM tool used to simulate particle interactions in shaping and gripping processes [4,25].
- MercuryDPM: Open-source library for numerical simulation of particle interactions and visualization of bulk processes.
- LAMMPS: Used for simulations of steady-state friction and protocol-dependent jamming of spherical particles [33].
- EDEM: A specialized tool for solving particle mass, mentioned in sources as a particle pressure solver on membrane boundaries in combination with ABAQUS [30].
- Other benchmarked DEM tools: Tools such as Blaze-DEM, GranOO, MFiX, MUSEN, Yade, and ESyS-Particle were compared in an extensive comparative study by the authors [27].

An analysis of available open-source DEM simulation frameworks showed that each software can be used to solve the proposed case studies. It was shown that simulating the same initial setup leads to similar results in almost all cases [27]. At the end of 2025, a new module called the Granular Flow Module was added to Comsol Multiphysics v. 6.4, which responds to the need for the accurate numerical modeling of systems using the DEM method. The basis of the module is the calculation of collision and contact forces between particles and between particles and the walls of the model domain. Standard contact models (e.g., the Hertz–Mindlin model) are implemented, which allow for the consideration of normal and tangential forces, friction, the coefficient of restitution, rolling resistance, or possible adhesion. Although the module was primarily developed for industrial applications such as emptying containers, material flow in silos, powder mixing, or blockage analysis, it has the potential to extend to other areas [34]. The disadvantage of this module is that it is not yet fully implemented into the structure of multiphysics modules. Since this module was only made available at the end of 2025, it is not yet possible to make any comparisons with other DEM simulation tools. Its potential uses in the field of soft robotics are still at the beginning, although with prospects, especially when using granular jamming.

(c) Hybrid and optimization frameworks: Since the granular core and shell exhibit different mechanical properties, it is necessary to include both components in the modeling procedure to accurately predict the behavior of the structure [5]. Modern research is directed towards integration of the DEM and FEM, which allows the modeling of nonlinear interactions between the membrane boundaries and the grains [1,3]. The FEM is used to accurately describe the deformations of the chamber due to (external) loading. Furthermore, for small particle numbers, the DEM can describe particle-level interactions in granular materials with low computational resource consumption compared to the traditional FEM [27]. Furthermore, evolutionary algorithms (EAs) in conjunction with DEM simulations allow for the in silico optimization of grain and membrane morphology for specific tasks [4,9,22].

- ProjectChrono: An open multiphysics engine used to study locomotion, control, and scalability of modular soft robots [2].
- Chrono: SolidWorks 2024: Add-on module for integrating CAD models into dynamic simulations [2].
- Kratos Multiphysics: Identified as a multiphysics FEM framework that also supports DEM simulations and their interconnection [25].

Regarding the bonding strategy in jamming systems, the key differences are in how the different technologies transfer forces and achieve stiffness changes. These strategies can be divided into surface bonding (characteristic of layered jamming) and bulk bonding (typical of granular jamming), with the degree of this bonding determining the transition between the weak (unjammed) and strong (jammed) states [10].

Weak vs. Strong Bonding (Phase Transition)

- Unjammed state: In this state (without vacuum or at ambient pressure), the particles or layers behave like a fluid. The particles are free to move and have low viscosity and minimal resistance to deformation, allowing the system to adapt to complex object geometries.
- Jammed state: By applying vacuum or mechanical compression, the particles/layers are forced together, creating mechanical interlocking and drastically increasing interparticle friction [10]. Force chains are formed, which give the aggregate the rigidity of a solid [4,15].

Surface bonding—this strategy is dominant in laminar jamming. It is based on overlapping flat sheets or strips of material [16]. The forces are thus distributed over a large contact area, which allows high stiffness to be achieved at relatively low pressures [6].

Volume bonding—this approach is the core of granular jamming. Stiffness results from the cooperative behavior of the entire set of particles in a closed volume [10].

(d) Auxiliary calculation and modeling tools

- MATLAB R2024a: Widely used for the identification of force transmission parameters, experimental data processing [35], image analysis (DIC), and trajectory tracking [10,36].
- FreeCAD 1.0.2: Used in conjunction with scripting to automatically generate CAD models from genetic algorithms for in materio membrane evolution [9].
- Python 3.13 (library LMFIT): Used for nonlinear identification of material constants in constitutive models [5].

### 2.3. Emerging Possibilities

New trends in granular jamming extend its applicability beyond traditional universal grippers:

- Magnetic Granular Jamming (MGJ): Provides a wireless alternative to pneumatic actuation. It uses external magnetic fields to induce attractive forces between ferromagnetic particles, allowing for an extremely fast response (under 0.1 s) and precise linear stiffness control without the need for tubing [21,31]. In [6], the authors designed a soft gripper using a dry ferromagnetic material.
- Active fluidization and vibration: The integration of vibration elements (e.g., audio drivers) enables the active release of accumulated stress during the grip formation process, leading to better object girth and higher retention force [4,11,27].
- Advanced fabrication and 3D printing: The use of multi-material 3D printing enables the production of bespoke granules with super-quadratic shapes and membranes with pre-programmed deformations, optimizing performance for specific object geometries [3,4,37]. The significance of the transition lies in the fact that natural grains exhibit uncontrolled changes in shape and size, while artificial grains allow precise control of the morphology and size of their components [4].
- New geometries and hybrids: Designs such as the “Jamming Donut” (torus actuator) enable “free-space” gripping, which is crucial for automated fruit picking. Hybrid systems combining granular jamming with chain structures [31] or honeycomb structures increase directional stability and load capacity [4,14]. New hybrid systems combining granules and a rigid chain structure are also being developed to increase the average stiffness change in all directions and the maximum force in a specific direction [38].

Advances in simulations and materials engineering are transforming granular jamming from an experimental concept to a reliable technology for medical rehabilitation (exoskeletons) [10,31], underwater manipulation, and safe collaborative robotics [3].

## 3. Applications in Soft Robotics

An analysis of published sources confirms that jamming technology (especially granular) has established itself as a key mechanism for variable stiffness in soft robotics [8,39]. Granular jamming is commonly used in the design of universal grippers to grip a wider range of objects compared to traditional joint-based robotic grippers. In the field of wearable technologies, granular jamming is mainly used to modulate joint stiffness and support weight-bearing limbs [40]. It is also used to design a rigid head mount for safer image-guided operations [41,42].

Although the following applications demonstrate the functional versatility of granular jamming in soft robotics, their development is heavily dependent on appropriate numerical simulation frameworks. In most reported cases, the DEM is used to understand the particle-level mechanisms that control stiffness transitions, FEM supports structural design at the actuator and system levels, and hybrid FEM–DEM approaches are used when the accurate prediction of membrane-grain interaction and nonlinear feedback effects is required. The choice of simulation strategy is therefore inherently application-driven.

This analysis also represents an evolving line of research moving from simple universal actuators to multilayer and hybrid systems with actively controlled stiffness.

1. Gripping Manipulation

This area remains the most dominant application, with modern technologies increasing the use of different approaches to gripper design.

Universal Grippers: Soft robotic grippers have emerged as a promising alternative to rigid manipulators in scenarios requiring safe and adaptive interaction with unstructured or fragile objects [8]. The work [43] represents a significant design step forward, using a three-layer arrangement, when an air interlayer is inserted between the rigid shell and the granular core. This concept represents a transition from purely passive jamming to an active modulated configuration. This concept is followed by the work [44], where performance improvement is achieved by structural modification of the layers. It presents multiple gripping modes within a single device. The granular mechanism is part of a broader architecture that allows switching between different mechanical gripping strategies. Thus, we are developing towards expanding the functionality of the system. The works [45,46,47] represent another quantitative shift: the principle of granular jamming is no longer applied exclusively to static gripping of an object but is integrated into moving structural elements. Compared to previous works, the purpose of use here changes—from object manipulation to the control of dynamic movement and interaction with the environment.

From a simulation frame, universal grippers represent one of the most extensively modeled applications of granular jamming. DEM-based simulations are primarily used to analyze particle rearrangement, packing density evolution, and force chain formation during object encapsulation. FEM models, in contrast, are typically employed to predict membrane deformation and global stiffness modulation under vacuum pressure. For the high-fidelity prediction of gripping force and hysteresis under cyclic loading, hybrid FEM–DEM frameworks are increasingly adopted, as they allow direct coupling between particle-scale interactions and membrane mechanics.

- Actuators: The jamming principle can also be achieved using pneumatic actuators, where different layers in the actuator can be activated to achieve different final stiffnesses [46,48]. In [35], a pneumatic actuator with variable stiffness is presented, which is able to elongate or stiffen. Unlike earlier solutions, this is no longer an additional stabilizing element but an actively controlled movement parameter. This concept is further developed in [37], where the authors propose a hybrid composite structure consisting of a driving layer and a damping layer. The driving layer with an arcuate air chamber aims to achieve large bending deformation. A membrane containing particles is integrated with the driving layer to modulate its stiffness, with experimental verification of a new actuator stiffness design under different pressures for use in the field of rehabilitation and intelligent automation [49].

Innovative designs include a soft cylindrical pneumatic actuator that can simulate the movements of living organisms, including the bending of finger joints [2], or a tubular actuator [18]. In the field of rehabilitation and assistive systems, the incidence of finger-attached actuators is the highest, as the devices are designed to be directly fixed to the individual fingers of the patient. We can find designs of a five-finger glove described in [29] and a four-finger hand [50]; in [35], soft robotic fingers are presented that integrate layered and granular locking into a single actuator, and a two-chamber finger is described in [8]. The laminar locking of robot joints is used in the design of a three-finger gripper [17].

In the field of actuator development, continuum models based on the finite element method (FEM) dominate the design process. However, near the jamming transition, continuum approximations tend to smooth out the discrete nature of particle rearrangements. For this reason, DEM simulations are often used in the early design phase to optimize particle morphology and size distribution, while hybrid FEM–DEM approaches are needed when the accurate modeling of stiffness evolution and cyclic durability is critical, such as in rehabilitation devices and assistive gloves.

2. Agriculture and Fruit Harvesting: Sources cite specific applications such as the “Jamming Donut” for open-field fruit harvesting [51] and Kirigami grippers for delicate food handling [27].

In this area, simulation frameworks play a crucial role in balancing adaptability and load capacity. Reduced-order FEM models are typically used to optimize global geometry and weight, especially when integration into robotic arms is required. DEM simulations, on the other hand, support the optimization of grain properties to ensure sufficient holding force without damaging delicate produce. Hybrid approaches become particularly relevant when interaction with irregular biological surfaces must be accurately captured.

3. Aerial handling: Systems are emerging such as TRIGGER, a lightweight universal gripper for drones, capable of exerting a holding force of up to 15 N [4]. Here, it is shown that weight reduction and stiffness optimization enable the use of jamming in mobile platforms with limited load capacity.

The FEM is typically used in this area to minimize mass while maintaining stiffness tunability. DEM simulations contribute to optimizing the behavior of the granular core, especially when evaluating the holding force with respect to the vacuum level. Although hybrid models are computationally demanding, they are used to verify safety margins in lightweight drone-mounted grippers.

4. Underwater manipulation: However, water can also be used as a liquid medium to control granular interference, which is used for the design of grippers working underwater [4]. The authors in [52] present a layered jamming-based tentacle that exhibits effective adhesion underwater. Furthermore, hydraulic jamming can be used for underwater archaeology and sample collection at depths where high ambient pressure increases the effectiveness of the grip [53].

For applications of this type, FEM-based simulations are essential due to the multi-physics complexity inherent in the underwater environment. DEM contributes to the understanding of particle compaction at an elevated ambient pressure, and hybrid frameworks allow for the simultaneous consideration of all aspects.

5. Wearable robots that use jamming technology to achieve variable stiffness and safe human interaction—for limb exoskeletons [10,14,54]. This trend represents a functional shift from object manipulation to biomechanical human support.

FEM simulations are typically used to model structural compliance and human–robot interaction forces, while DEM-based analyses provide insight into internal force transmission in the granular core. Hybrid frameworks are used to bridge the gap between the simulation and experiment in exoskeleton development.

The overall analysis of the literature thus shows a clear line of development:

1. Simple vacuum universal grippers—emphasis on adaptability;
2. Multilayer and multimodal constructions—emphasis on control and variability;
3. Integration into actuators and motion elements—emphasis on dynamic force;
4. Hybrid and application-specific systems—emphasis on optimization for a specific domain.

Granular jamming is thus evolving from an experimentally demonstrated universal gripping principle to a comprehensive technological platform for stiffness control in a wide range of soft robotic systems.

## 4. Comparative Synthesis of Simulation Frameworks

The reviewed literature shows that the choice of a numerical simulation framework for granular jamming in soft robotics is strongly application-dependent and reflects a trade-off between computational efficiency and physical accuracy. Based on the sources considered, a wide range of simulation tools can be identified for modeling granular jamming mechanisms, membrane deformations, and particle interactions. These tools are primarily divided into finite element method (FEM) and discrete element method (DEM) software. The fundamental principles and implementation details of FEM, DEM, and hybrid FEM–DEM simulation frameworks are described in Section 2.2. This section therefore focuses on their comparative performance, limitations, and design implications. In terms of the use of the described simulation frameworks, the following results emerge:

- From a design methodology perspective, DEM-based simulations play a critical role during the early conceptual phase [4]. Because stiffness modulation in granular jamming is governed by particle rearrangement and force chain formation, the DEM is indispensable for selecting particle morphology, size distribution, and material properties. However, its unfavorable computational scaling restricts its use to localized studies rather than full-system simulations.
- In contrast, FEM-based models dominate the actuator- and system-level design stages. By representing the granular core as an effective continuum, FEM enables efficient evaluation of membrane deformation, global stiffness modulation, and structural integrity under external loading. This approach assumes a smooth and often isotropic evolution of stiffness as the vacuum level changes. In reality, however, the jamming transition is driven by discrete particle rearrangements and the sudden formation of force chains within the granular system. Since the FEM does not explicitly distinguish individual particles, these discontinuous and heterogeneous processes are replaced by continuous constitutive relations. As a result, the transition from unjammed to jammed states appears numerically smoothed. This smoothing can lead to an underestimation of stiffness gradients and a reduced ability to capture hysteresis under cyclic loading. This aspect is more comprehensively treated in the literature [55].
- Hybrid FEM–DEM frameworks bridge this gap by explicitly coupling particle-scale dynamics with membrane deformation. Unlike standalone approaches, hybrid models are capable of capturing nonlinear feedback mechanisms between grains and elastic boundaries, including hysteresis effects under cyclic loading. As a result, they provide the highest fidelity among current simulation strategies and significantly reduce the simulation–experiment discrepancy reported in the literature. Despite these advantages, hybrid approaches remain computationally demanding and sensitive to numerical coupling strategies, limiting their widespread adoption.

This approaches can be categorized according to their coupling strategy.

- In one-way (weak) coupling schemes, particle-scale forces computed in the DEM are transferred to the FEM membrane model without iterative feedback. This approach reduces synchronization overhead and offers a moderate computational cost increase (typically 2–5× compared to standalone FEM) but may underestimate nonlinear feedback effects near the jamming transition.
- In two-way (strong) partitioned coupling, force and displacement fields are exchanged iteratively within each time step. This improves accuracy in capturing membrane–particle interactions and stiffness evolution but increases the computational cost significantly (often 10–50× relative to FEM), particularly due to convergence requirements.
- Monolithic coupling formulations solve particle and continuum equations within a unified system matrix. While offering the highest numerical consistency and stability, this approach is computationally the most demanding and typically restricted to reduced-scale systems or high-performance computing environments.

The selection of a coupling strategy is therefore application-dependent: weak coupling may be sufficient for preliminary actuator design, whereas strong or monolithic coupling becomes necessary when the accurate prediction of hysteresis, cyclic loading, or stiffness gradients near the jamming transition is required.

To provide a structured overview of the fundamental differences between these simulation frameworks, Table 1 summarizes their characteristic levels of detail, computational complexity, modeling limitations, and fidelity to real-world behavior.

A structured comparison of the modeling capabilities, advantages, and limitations of the investigated simulation approaches is summarized in Table 2, which summarizes the findings from the analyzed literature and shows their typical areas of applicability in soft robotics.

Jamming technology has established itself as a dominant mechanism for variable stiffness in soft robotics, allowing for transitions between liquid and solid states with a minimal volume change [1,13]. The main trend is the transition from simple bag-like grippers to complex morphologies (e.g., Kirigami, bellows structures) and hybrid systems that combine granular jamming with layered jamming or mechanical joints to achieve higher load capacity and precision [10,14].

### 4.1. Workflow for Selecting a Simulation Framework

**Figure 5:**
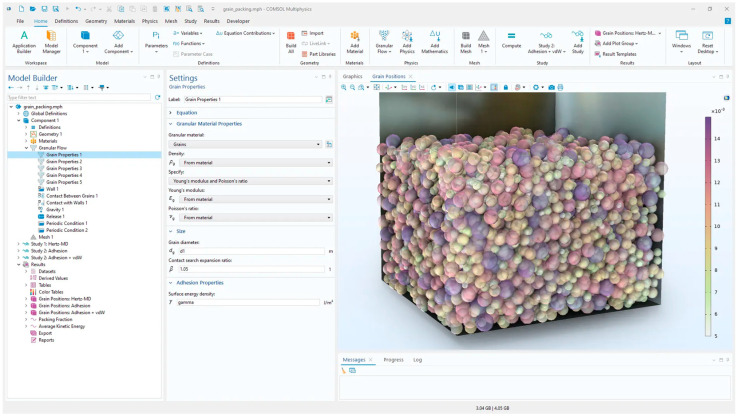
Settings of granular flow in Comsol Multiphysics 6.4 [34].

An analysis of the available resources has shown that no numerical approach is universally optimal for modeling granular jamming systems in soft robotics. Instead, the choice of a simulation framework strongly depends on the modeling goal, the desired physical accuracy, the scale of the system, and the available computational resources. To support a systematic and transparent selection of an appropriate simulation framework, a decision-making workflow is proposed and summarized in Figure 5.

The presented workflow also includes practical constraints such as the system scale and computational resources. For small subsystems or low particle numbers, the DEM remains feasible, while full actuator or gripper simulations typically require FEM or simplified hybrid strategies. By integrating modeling objectives, physical relevance, and computational feasibility, the workflow provides structured guidance for selecting an appropriate simulation framework at different stages of soft robotic system development.

### 4.2. Challenges and Future Prospects

From a materials research perspective, sources confirm that grain properties (shape, size, stiffness) are crucial for the resulting performance; for example, soft particles (EPS) significantly improve the encapsulation of objects, while stiff particles increase mechanical interlocking [4,56,57]. The future of the field is towards bespoke (tailor-made) particles manufactured through 3D printing, which allow for optimizing the packing density and retention force for specific tasks [3,4,38].

**Figure 6:**
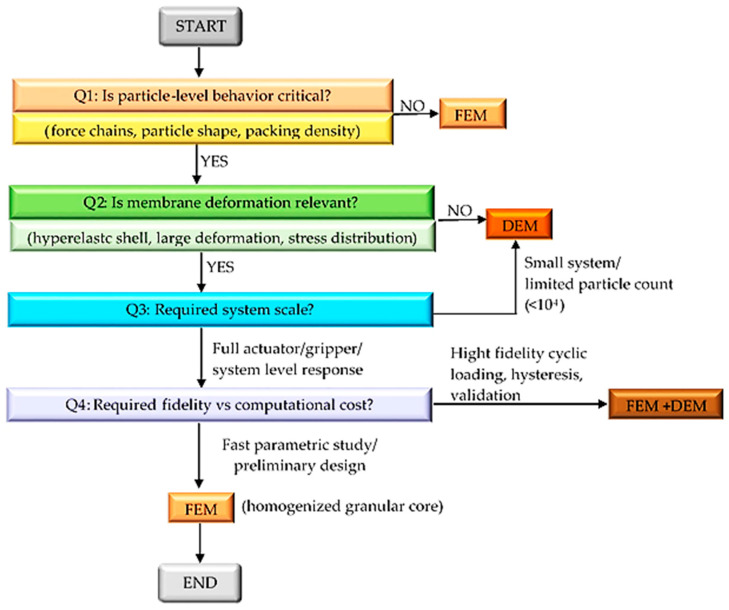
Workflow for selecting an appropriate framework.

The critical point remains the computational modeling of nonlinear interactions. The implementation of advanced simulation frameworks, in particular the coupling of the DEM (for particles) and FEM (for membranes), is becoming essential for predicting the behavior of systems under cyclic loading and for eliminating the so-called “reality gap” in design [1,5,9,25]. Figure 6 summarizes the key limitations of current simulation frameworks and the anticipated directions for their future development.

**Figure 7:**
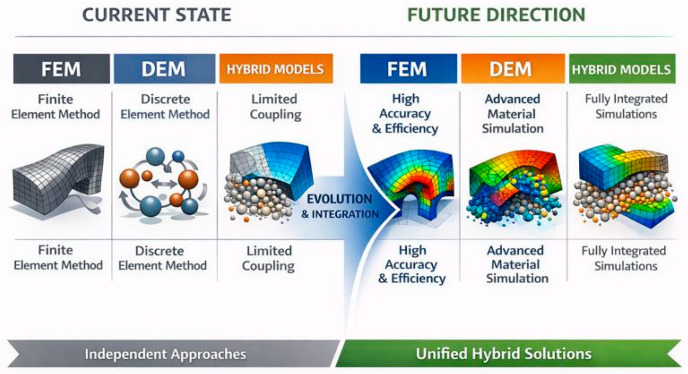
Development outlook of simulation frameworks for granular jamming in soft robotics.

A review of the literature suggests that hybrid FEM–DEM frameworks (Figure 7) are likely to dominate future developments, but only if significant progress is made in computational efficiency and adaptive modeling. At the same time, it is expected that machine learning [4,58], genetic algorithms for finding the optimal membrane and grain morphology [9], and purely physical models will be increasingly complemented by data-driven approaches, especially in optimization and control tasks.

Table 3 represents the assumptions for the development of granular jamming and soft robotics.

From the point of view of soft robotics (Table 4), the possibility of using magnetic jamming appears to be the most significant. It provides the prerequisites for reducing the dependence of soft robots on external air distribution. It presents possibilities for the design of self-sufficient systems that can be controlled by a remote magnetic field, which is extremely beneficial for biomedical applications and surgery [21,36].

## 5. Conclusions

The presented paper maps the current state of knowledge in two critical domains: simulation frameworks and new technological possibilities. In the area of modeling, it is possible to observe the increasing importance of the coupling of the discrete element method (DEM) for particle simulation and the finite element method (FEM) for membrane modeling, which are essential for eliminating the so-called “reality gap” in the design of complex actuators [4,5,36].

From the analysis of the literature in the field of modeling, it is clear that no single simulation approach is sufficient to fully capture the complex behavior of granular fault systems. Discrete element methods are essential for understanding particle-level interactions and force chain formation, while finite element methods are essential for accurately modeling membrane deformation and structural responses. As a result, hybrid FEM–DEM frameworks are increasingly in the spotlight as the most promising solution for realistic system-level modeling, despite their high computational cost and implementation complexity.

In parallel, we explore emerging trends that exceed the limits of traditional pneumatic jamming, in particular magnetic granular jamming (MGJ) with ultrafast response times below 0.1 s, mechanisms using positive pressure, and advances in the field of 3D printed particles with a precisely defined morphology [20,29,54].

Further developments can be expected in the following areas:

- hybridization—combining granular jamming with pneumatic systems, magnetism, or layered jamming [1,2,4];
- multiphysics modeling—linking the behavior of fluids, particles, and membranes in real time [1,4,25];
- bekspoke design—creating specialized grains and membranes tailored to specific tasks through 3D printing [3,38].

However, significant challenges remain, particularly in reducing computational demands, improving model validation, and bridging the gap between simulation and real-world performance. Future research should therefore focus on developing efficient hybrid and multiphysics models, standardized validation benchmarks, and application-specific simulation strategies that will enable reliable digital twins of soft robotic systems utilizing granular jammings.

## Figures and Tables

**Table 1:** Summary of strategies.

Property	Surfer Layer (s)	Bulk Layer (Granules)
Primary source of force	Interfacial friction [6,16]	Interparticle friction and bulk interlocking [10]
Adaptability	Limited to 2D bending [30]	High—adapt to 3D shapes [9]
Flexural stiffness	Significantly higher per unit weight [16,17]	Lower—prone to deformation under load [14,37]
Compactness	Compact, low profile	Bulk—requires space for granules
Typical applications	Surgical arms, exoskeletons [16]	Universal grippers, robotic feet

**Table 3:** The frequency of use of individual simulation frameworks.

Method	Modeled Phenomena	Key Advantage	Limitations	Typical Application
FEM	˗ Large deformation of compliant membranes ˗ Hyperelastic and viscoelastic material behavior ˗ Global stress–strain response ˗ Pressure-driven stiffness change (continuum approximation)	˗ Computationally efficient for large structures ˗ Well-established numerical framework ˗ Accurate modeling of soft materials and boundary conditions ˗ Suitable for parametric studies and optimization	˗ Granular media represented as homogenized continuum ˗ Inability to capture force chains and particle rearrangement ˗ Limited accuracy near jamming transition ˗ Requires strong simplifying assumptions	˗ Structural design of soft actuators ˗ Membrane and chamber optimization ˗ Preliminary design studies ˗ Fatigue and durability analysis
DEM	˗ Particle–particle contact ˗ Frictional interactions and force chains ˗ Particle rearrangement and packing density ˗ Jamming/unjamming transitions at particle scale	˗ High physical fidelity at micromechanical level ˗ Direct modeling of particle shape and material ˗ Insight into fundamental jamming mechanisms ˗ Suitable for studying granular core behavior	˗ High computational cost for large particle counts ˗ Limited ability to model soft membranes ˗ Not suitable for full system simulations ˗ Requires careful contact model calibration	˗ Optimization of granular media ˗ Particle shape and size studies ˗ Analysis of force transmission ˗ Fundamental jamming research
FEM-DEM	˗ Coupled membrane deformation and particle interactions ˗ Particle–structure contact ˗ Pressure-dependent stiffness evolution ˗ Nonlinear system-level response	˗ Most realistic representation of granular jamming systems ˗ Captures both macro- and micro-scale behavior ˗ Reduced simulation–experiment gap ˗ Enables application-driven design	˗ Very high computational cost ˗ Complex coupling and implementation ˗ Limited scalability ˗ Challenging parameter tuning and validation	˗ Universal grippers ˗ Adaptive stiffness devices ˗ Cyclic loading and durability studies ˗ Digital twin development

**Table 4:** The assumptions for the development of granular jamming and soft robotics.

Category	Key Assumption/Direction	Significance and Expected Impact
Simulation frame	Coupling DEM and FEM	Essential for accurate modeling of nonlinear interaction at the granule-membrane interface; simulation of complex deformations [4,31].
	Transition from spherical to superquadrilateral grains	Modeling non-spherical shapes (e.g., ellipsoids) in simulations reduces the “reality gap” and allows for predicting force chains [1,4,26,39]
Materials research	Use of soft and deformable particles	Soft particles (e.g., EPS) allow for better encirclement of objects and increase static friction in a jammed state [4,10,39].
	Development of bespoke grains through 3D printing	Precise control over particle morphology and texture allows for optimizing retention power for specific tasks [4,10,39].
Drive technologies	Magnetic Granular Jamming (MGJ)	It allows for wireless (untethered) activation and super-fast response without the need for heavy vacuum pumps [21,36].
	Active fluidization using vibrations	Use of resonant modes to release granule tension; increases contact area and grip stability [12,38]
Management and control	Sensor integration for feedback	Implementation of thin-film FSR sensors or optical sensing for real-time adaptive gripping strategies [5,21].
Design methodology	Standardization of performance metrics	The need for unified benchmarks for measuring stiffness and strength to objectively compare different jamming systems [1,26].
Construction	Hybridization of jamming mechanisms	Combination of granular, layered, and fiber jamming to increase bending load capacity and movement accuracy [1,3,30,37,53]

**Table 2:** Comparative overview of FEM, DEM, and hybrid FEM–DEM frameworks in granular jamming.

Parameter	FEM	DEM	Hybrid/Combined Approach
Level of detail	Macroscopic (continuum) [5,13]	Microscopic (individual grains) [4,26]	Multiphysics (grain + shell) [4,31]
Main domain	Deformation of membranes, soft finger bending, haptics [27,43]	Grain physics, force chains, friction, breakage [20]	Grain-membrane interaction, robotic locomotion [2]
Typical particle count	Not particle-resolved	10^3^–10^5^ particles (CPU); up to ~10^6^ with HPC/GPU	Typically limited to 10^3^–10^4^ particles due to coupling overhead
Computational complexity	Medium to high (depends on remeshing)	Very high (at >100k particles) [26]	Extremely high (solver synchronization)
Main limits	Often simplifies the kernel to an isotropic continuum [10,13]	Problem with modeling elastic boundaries of membranes	Implementation complexity and hardware requirements (GPU/CPU) [26]
Stiffness prediction near jamming transition	May smooth transition; underestimation of stiffness gradient	Accurate capture of force-chain formation	Highest fidelity; captures nonlinear feedback
Typical stiffness prediction deviation near transition	~10–30% deviation depending on constitutive assumptions	<10% if contact model calibrated	Comparable to DEM; reduced simulation–experiment gap
Fidelity to reality	Good for linear/hyperelastic behavior	Excellent for bulk materials physics	Highest—eliminates the “reality gap” in design [4]
Hardware requirements	Standard workstation	High RAM; multi-core CPU or GPU recommended for >10^5^ particles	HPC or GPU strongly recommended

## Data Availability

Not applicable.
